# Cross-disease transcriptomic meta-analysis and network pharmacology reveal key therapeutic targets in rheumatoid arthritis, systemic lupus erythematosus and multiple sclerosis

**DOI:** 10.3389/fbinf.2025.1744094

**Published:** 2026-01-21

**Authors:** K. Lakshmi, Sundararajan Vino

**Affiliations:** Integrative Multiomics Lab, Department of Bio Sciences, School of Bio Sciences and Technology, Vellore Institute of Technology, Vellore, Tamil Nadu, India

**Keywords:** autoimmune diseases, differentially expressed genes, functional enrichment analysis, molecular dynamics, transcriptome

## Abstract

Autoimmune disease has a complex etiology that remains not fully understood. We aimed to identify highly perturbed DEGs and hub genes associated with autoimmune disease Rheumatoid Arthritis (RA), Systemic Lupus Erythematosus (SLE) and Multiple Sclerosis (MS). To find potentially lead to more effective therapies that target the root causes of these diseases.

**Materials and methods:**

Datasets for autoimmune diseases (RA, SLE, and MS) were collected from the GEO database. Differentially expressed genes were identified and subjected to meta-analysis to obtain common DEGs, which were then used for functional enrichment analysis GO and pathway analysis. A PPI network was constructed, and topology-based ranking identified hub genes. These hub genes were further analyzed through regulatory network analysis (TF and miRNA), gene-disease association studies, and drug-gene interaction analysis. Finally, molecular docking and molecular dynamics (MD) simulations were performed on the hub genes.

**Results:**

A total of 341 differentially expressed genes were identified, with 172 upregulated and 169 downregulated genes. Among these, eight hub genes *STAT1, PTPRC, IRF8, JAK2, IL10RA, OAS2, CCR1*, and *IFI44L* were found to be closely associated with the disease. Functional enrichment analysis revealed significant involvement in 143 biological processes, 53 cellular components, and 67 molecular functions, as well as 60 KEGG pathways. Further regulatory network analysis highlighted the interactions of the suggested hub genes with 198 transcription factors (TFs) and 993 microRNAs (miRNAs). Additionally, these genes were associated to 2,769 diseases, and 132 drugs were identified to interact with them. Molecular docking studies, along with Molecular Dynamics Simulation (MDS) stability analysis, demonstrated the potential of natural compounds and known immunomodulatory drugs as promising therapeutic targets for clinical application.

**Conclusion:**

These findings explored identifying the DEGs among shade of the autoimmune disease RA, SLE, MS, and this hub gene are associated with transcription factors are most crucial role play in the disease potentially clinical therapeutic targets of the autoimmune disease.

## Introduction

Autoimmune diseases (AIDs) such as rheumatoid arthritis (RA), systemic lupus erythematosus (SLE), and multiple sclerosis (MS) are chronic, multifactorial conditions characterized by immune system dysregulation and sustained inflammation. Although these diseases exhibit distinct clinical manifestations. RA primarily affects synovial joints, SLE involves multi-organ damage, and MS targets the central nervous system, they share overlapping pathogenic mechanisms including the activation of innate and adaptive immune responses, cytokine overproduction, and autoantibody formation (Song et al., 2025; Frazzei et al., 2022; Marrie et al., 2015). Recent transcriptomic evidence highlights the involvement of type I interferon (IFN-I) signaling as a central immune modulator across these autoimmune conditions (Rose et al., 2013; Lerkvaleekul et al., 2022; Guo M. et al., 2024).

Siglec-1 (sialic acid-binding Ig-like lectin-1, CD169), a monocyte/macrophage-specific surface receptor, is a well-characterized IFN-I-inducible gene (Macauley et al., 2014; Brzezicka and Paulson, 2023; Biesen et al., 2008). Unlike most other Siglecs, Siglec-1 lacks immunoreceptor tyrosine-based inhibitory motifs (ITIMs), but mediates key immunomodulatory functions via adhesion and endocytic roles (Macauley et al., 2014; Zheng et al., 2015). Elevated Siglec-1 expression has been reported in RA, SLE, and MS patients and correlates strongly with clinical activity indices such as DAS28 and SLEDAI, as well as with biomarkers including CRP and anti-dsDNA antibodies (Xiong et al., 2014; Lim et al., 2018; Oliveira et al., 2018; Biesen et al., 2008; Stuckrad et al., 2020). In MS, Siglec-1-positive myeloid cells are enriched in active brain lesions, implicating their role in acute neuroinflammation (Ostendorf et al., 2021).

Given the central role of IFN-I and its downstream effectors in AID pathogenesis, there is an urgent need to identify convergent transcriptomic signatures and molecular drivers that transcend individual disease boundaries. While prior studies have investigated DEGs within isolated disease contexts, few have integrated gene expression profiles across RA, SLE, and MS in a unified systems biology framework (De Silva et al., 2022; Cheng et al., 2024; Sun et al., 2014).

**FIGURE 1:**
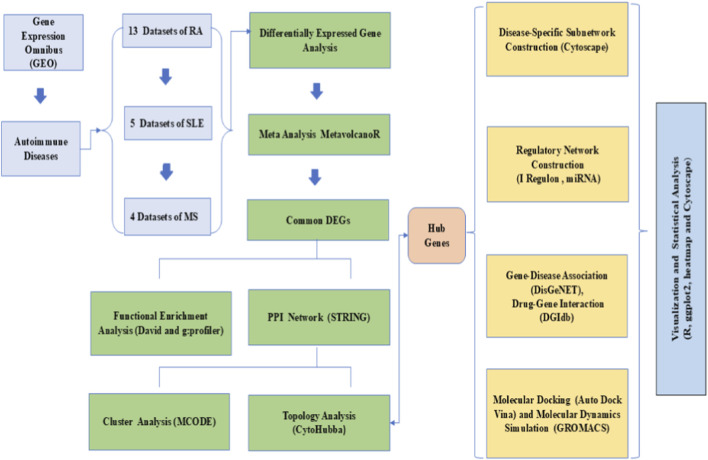
Overall workflow of the study.

In the present study, we performed a large-scale meta-analysis (Table 1) of publicly available transcriptomic datasets to identify differentially expressed genes shared across RA, SLE, and MS. The overall workflow is depicted in Figure 1. Functional enrichment analyses, protein-protein interaction (PPI) network construction, transcription factor and microRNA regulatory mapping, and drug-gene interaction analyses were performed to characterize core molecular networks. We also used molecular docking and dynamics simulation to validate the draggability of selected hub proteins, comparing natural compound interactions to known immunomodulatory drugs. Our findings highlight several key immune regulators, including *STAT1, JAK2*, and *OAS2* as potential therapeutic targets, alongside Siglec-1, providing a comprehensive resource for the development of broad-spectrum therapeutics in autoimmune disease management.

**TABLE 1:** Summary of tools, parameters and software in this study.

Process step	Tools/database	Parameters	Software version
Differential expression gene	GEO2R, Limma	p-value <0.05 and logFC value >1	R.4.5.1
Meta analysis	MetavolcanoR	p-value <0.05 and logFC value >1	R 4.5.1
Functional enrichment analysis	DAVID and g: Profiler.	p-value <0.05	*e113_eg59_p* *19_f6a03c19*
PPI network and MCODE clusters.	STRING database, cytoscape	Confidence score >0.40	11.5 3.10.2
Hub gene identification	Cytoscape (CytoHubba)	Degree, closeness, MCC Top 10 in >3 metrics	3.10.2
Disease-specific subnetwork	STRING database, cytoscape	Confidence score >0.40	3.10.2
Regulatory network (TF)	iRegulon (TF), cytoscape	FDR ≤ 0.0010. Confidence score >0.40	1.3 3.10.2
Regulatory network (miRNA)	miRDB, cytoscape	Score >80	3.10.2
Gene-disease association	DisGeNET cytoscape	Confidence score >0.40	3.10.2
Drug-gene interaction	DGIdb cytoscape	Confidence score >0.40	3.10.2

## Methodology

### Data acquisition

We retrieved gene expression datasets for RA, SLE, and MS from the Gene Expression Omnibus (GEO), focusing on human studies that included both disease and control samples (Table 2). Inclusion criteria ensured consistent platform technologies (Affymetrix, Illumina), normalized data, and a minimum of 10 samples per group to ensure statistical robustness. GEO serves as a valuable resource for unbiased data mining and disease comparison across multiple conditions (Barrett et al., 2013).

**TABLE 2:** GEO dataset collection for autoimmune disease (RA, SLE, MS).

S. No	Accession ID	Disease	Platform	Sample type control/Disease	Reference
1.	GSE55457	RA	Affymetrix human genome U133A array	Tissue; 10/13	PMID: 24690414
2.	GSE55235	RA	Affymetrix human genome U133A array	Tissue; 10/10	PMID: 24690414
3.	GSE15573	RA	Illumina human-6 v2.0 expression beadchip	Blood; 18/15	PMID: 19710928
4.	GSE1919	RA	Affymetrix human genome U95A array	Tissue; 5/10	PMID: 20858714
5.	GSE36700	RA	Affymetrix human genome U133 array	Tissue; 4/7	PMID: 25927832
6.	GSE247226	RA	Illumina NovaSeq 6000	Tissue; 6/6	PMID: 38137409
7.	GSE89408	RA	Illumina HiSeq 2000	Tissue; 28/151	PMID: 28455435
8.	GSE17755	RA	Hitachisoft AceGene human oligo chip 30K 1 chip version	Blood; 112/45	PMID: 28863153
9.	GSE12021	RA	Affymetrix human genome U133A/B array	Tissue; 12/9	PMID: 18721452
10.	GSE77298	RA	Affymetrix human genome U133 plus 2.0 array	Tissue; 16/7	PMID: 26711533
11.	GSE100191	RA	Agilent-026652 whole human genome microarray 4 × 44K v2	Blood; 7/5	PMID: 29584756
12.	GSE93272	RA	Affymetrix human genome U133 plus 2.0 array	Blood; 78/43	PMID: 30013029
13.	GSE64612	RA	SABiosciences innate and adaptive immune responses PCR array	Blood; 40/20	PMID: 22238028
14.	GSE51997	SLE	Affymetrix human genome U133 plus 2.0 array	Blood; 6/4	PMID: 24391825
15.	GSE52471	SLE	Affymetrix human genome U133A 2.0 array	Skin; 13/25	PMID: 23771123
16.	GSE30153	SLE	Affymetrix human genome U133 plus 2.0 array	Blood; 9/17	PMID: 21886837
17.	GSE13887	SLE	Affymetrix human genome U133 plus 2.0 array	Blood; 9/10	PMID: 19201859
18.	GSE10325	SLE	Affymetrix human genome U133A array	Blood; 28/39	PMID: 18275831
19.	GSE21942	MS	Affymetrix human genome U133 plus 2.0 array	Blood; 15/12	PMID: 22021740
20.	GSE26484	MS	Affymetrix human genome U133 plus 2.0 array	Blood; 14/6	PMID: 22491253
21.	GSE23832	MS	Affymetrix human gene 1.0 ST array	Blood; 4/8	PMID: 21346816
22.	GSE16461	MS	Affymetrix human genome U133 plus 2.0 array	Blood; 8/8	PMID: 21216829

### Meta-analysis of gene expression

Differentially expressed genes (DEGs) for each dataset were identified using GEO2R, followed by integration using the MetavolcanoR package in R. This approach incorporates both p-values and fold-change data, generating consensus DEG lists across diseases. We applied a vote-counting method to accommodate inter-study variability. Genes were filtered using an adjusted p-value <0.05 and logFC value >1. This step enhances detection of consistent transcriptional changes across independent studies and increases the power to identify biologically relevant genes (Rau et al., 2014).

### Functional enrichment analysis

Common Differentially expressed gene were subjected to Gene Ontology (GO) and Kyoto Encyclopedia of Genes and Genomes (KEGG) enrichment analyses using DAVID and g: Profiler (version e113_eg59_p19_f6a03c19; https://biit.cs.ut.ee/gprofiler/gost). These tools identify overrepresented biological processes, molecular functions, and pathways such as interferon signaling and cytokine-mediated immune responses. Enrichment analysis contextualizes gene lists within established immunological frameworks (Huang da et al., 2009; Raudvere et al., 2019).

### Protein-protein interaction network and hub gene identification

Using the STRING database, we constructed high-confidence protein-protein interaction (PPI) networks of the DEGs. Cytoscape was employed to visualize these interactions. Hub genes were identified using CytoHubba (degree, MCC, and closeness centrality algorithms), while MCODE was used to detect densely connected clusters. Hub genes often represent master regulators or potential drug targets within disease-relevant networks (Szklarczyk et al., 2019; Chin et al., 2014).

### Disease-specific subnetwork construction

We constructed separate subnetworks for RA, SLE, and MS using disease-specific DEG lists. These were analyzed using STRING and visualized with Cytoscape to compare topological properties and key nodes. This step allowed us to detect both disease-specific regulators and shared molecular patterns across AIDs. Network metrics such as degree distribution, centrality, and clustering coefficient were compared to distinguish condition-specific versus overlapping hubs (Caldera et al., 2017).

### Regulatory network construction

We mapped transcription factors (TFs) and microRNAs (miRNAs) targeting the identified hub genes using Network Analyst, integrating data from iRegulon (TF) and miRDB (miRNA). Constructing these networks helped infer upstream regulatory mechanisms modulating autoimmune-related gene expression. These interactions reveal regulatory hierarchies and offer further therapeutic targeting options (Janky et al., 2014; Chen and Wang, 2020).

### Gene-disease association and drug-gene interaction

Gene-disease associations were retrieved from DisGeNET to validate the pathological relevance of identified hub genes. Drug-gene interaction predictions were sourced from DGIdb. Genes with known interactions with approved or investigational drugs were flagged as druggable targets. This integration aids in repurposing existing compounds and informs future therapeutic strategies (Piñero et al., 2020; Freshour et al., 2021).

### Molecular docking

We selected representative hub genes for *in silico* docking studies. Protein structures were downloaded from the Protein Data Bank (PDB), and ligands (including baricitinib, tofacitinib, luteolin, and quercetin) were sourced from PubChem. Auto Dock Vina (version 1.5.7) was used to compute binding affinities and pose predictions. Docking results were analyzed for binding energy and interaction residues. This approach provides an initial screen for potential therapeutic efficacy (Trott and Olson, 2010).

### Molecular dynamics simulation

The top-scoring protein-ligand complexes were subjected to 100 ns molecular dynamics (MD) simulations using GROMACS version software (2024). Simulations were conducted under standard physiological conditions. Root mean square deviation (RMSD), root mean square fluctuation (RMSF), and hydrogen bond stability were calculated to assess structural stability and binding retention. MD simulations validate docking predictions by modeling dynamic behaviour of biomolecular interactions (Abraham et al., 2015).

### Visualization and statistical analysis

Gene expression volcano plots, PPI, TF-miRNA regulatory, and drug interaction networks were visualized using R (ggplot2) and Cytoscape. All statistical analyses were performed in R, with a significance threshold set at p < 0.05. Data visualization facilitates intuitive understanding of complex results and highlights biologically significant patterns (Shannon et al., 2003; R Core Team, 2023).

## Results

### Identification of differentially expressed genes

**FIGURE 2:**
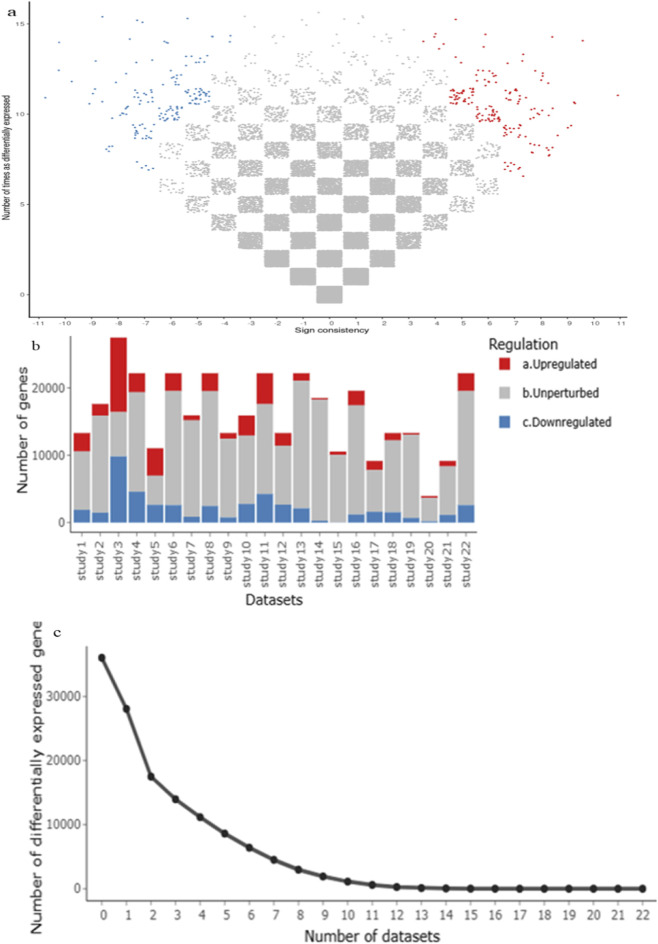
**(a–c)** A meta-analysis using the MetaVolcanoR package (vote-counting method) used to identify highly perturbed differentially expressed genes (DEGs), with upregulated genes shown in red and downregulate genes in blue.

A comprehensive meta-analysis across multiple GEO datasets for RA, SLE, and MS revealed 341 commonly dysregulated genes (Supplementary File). Of these, 172 genes were significantly upregulated and 169 were downregulated across all three disease conditions (Supplementary File). This high overlap underscores shared immune-inflammatory molecular signatures and validates the meta-analysis approach. The volcano plots (Figure 2a) illustrate consistent up- and downregulation across studies, while bar plots (Figures 2b,c) summarize gene counts per category. Notably, several DEGs such as *STAT1, OAS2*, and *IFI44L* appeared repeatedly across datasets, pointing to their conserved roles in autoimmune activation.

### Functional enrichment highlights interferon and cytokine signaling

**FIGURE 3:**
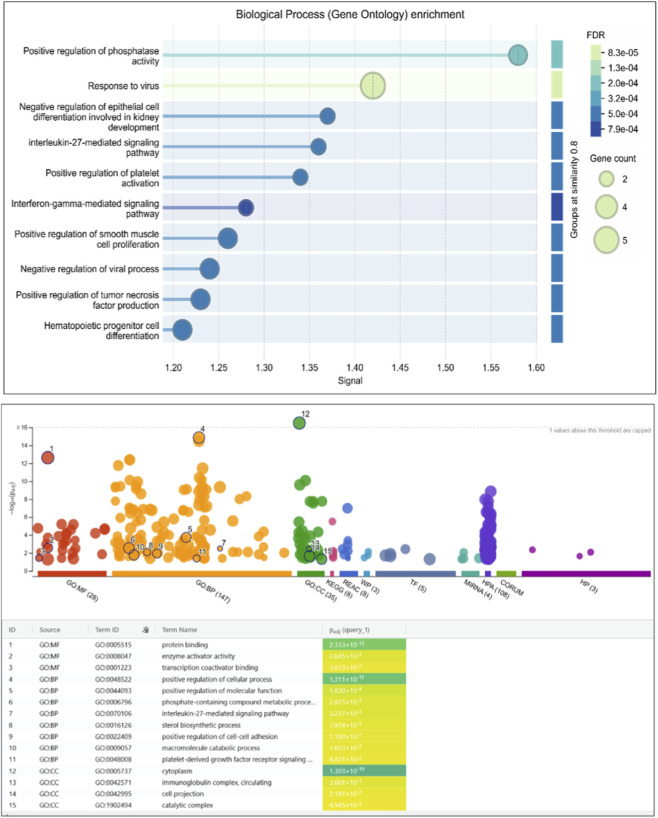
GO enrichment analysis of the common DEGs. Molecular function, biological processes, cellular component.

**FIGURE 4:**
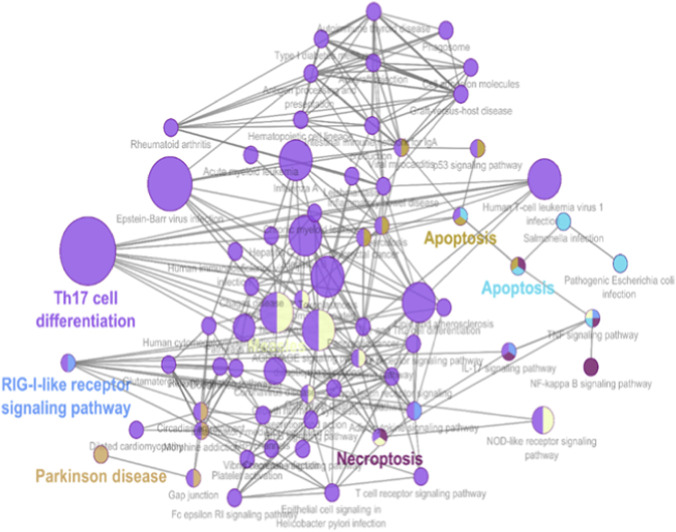
Pathway analysis KEGG pathways common DEGs.

GO and KEGG enrichment analyses were performed to determine the biological relevance of the 341 DEGs. These genes were highly enriched in biological processes related to immune system activation. Specifically, GO terms like “type I interferon signaling pathway,” “response to virus,” and “cytokine-mediated signaling” (Figure 3 and Supplementary File) dominated the enrichment profiles. KEGG analysis highlighted three major pathways: Toll-like receptor signaling, *Jak-STAT* signaling, and cytokine-cytokine receptor interaction, all of which are known to contribute to AID progression (Figure 4 and Supplementary File). These findings align with known IFN-I dysregulation in RA, SLE, and MS pathogenesis.

### Protein-protein interaction (PPI) network analysis and hub gene selection

**FIGURE 5:**
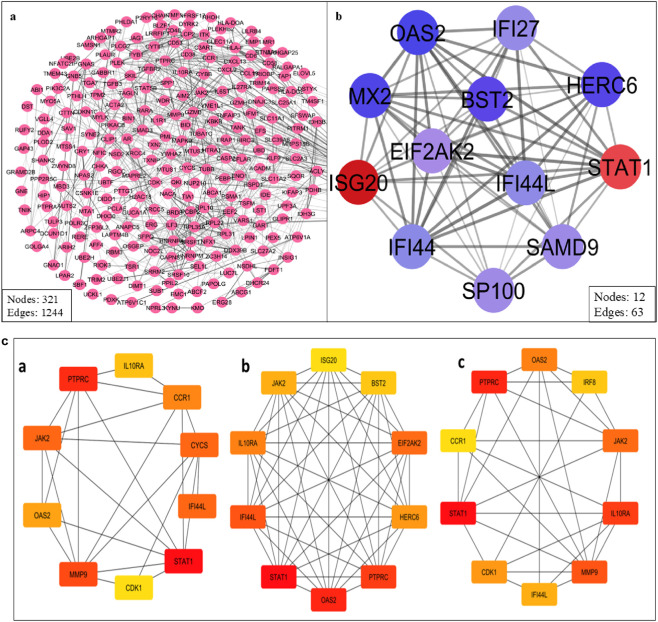
**(a)** PPI network of the common DEGs. **(b)** Cluster highly densely connected node. **(c)** The topological analyses of the PPI Network (a) Degree (b) MCC and (c) Closeness.

The STRING database was used to create a high-confidence (Figure 5a) PPI network of the common DEGs. This network was visualized and further analyzed in Cytoscape to identify (Figure 5b) densely connected nodes using MCODE, and topologically important genes using CytoHubba. Eight genes were identified as hubs: *STAT1, PTPRC, IRF8, JAK2, IL10RA, OAS2, CCR1*, and *IFI44L* (Table 3; Figure 5c). These genes showed the highest degree and MCC scores, suggesting central regulatory roles. Several of these, such as *STAT1* and *IRF8*, are known interferon-responsive transcription factors, while *IL10RA* and *CCR1* represent key signaling receptors.

**TABLE 3:** Topological analysis for Hub gene extracted three rank methods.

S. No	Degree	Closeness	MCC
1.	*STAT1*	*IL10RA*	*OAS2*
2.	*MMP9*	*IRF8*	*EIF2AK2*
3.	*IL10RA*	*MMP9*	*JAK2*
4.	*OAS2*	*IFI44L*	*PTPRC*
5.	*CDK1*	*OAS2*	*STAT1*
6.	*PTPRC*	*STAT1*	*IFI44L*
7.	*JAK2*	*PTPRC*	*ISG20*
8.	*IFI44L*	*JAK2*	*HERC6*
9.	*CYCS*	*CDK1*	*IL10RA*
10.	*CCR1*	*CCR1*	*BST2*

### Disease-specific subnetwork insights

**FIGURE 6:**
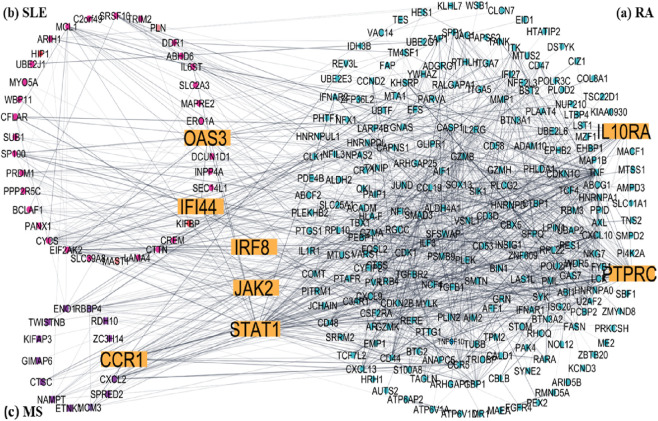
Disease-specific subnetworks construct of common DEGs Hub genes are yellow colour **(a)** RA, **(b)** SLE and **(c)** MS.

To understand disease-specific molecular patterns, DEG lists for RA, SLE, and MS were analyzed independently to construct condition-specific subnetworks (Figure 6). The RA subnetwork (Figure 6a) emphasized synovial inflammation genes such as *JAK2, IL10RA*, and *TNFSF10*. The SLE subnetwork (Figure 6b) revealed strong enrichment of IFN-stimulated genes including *IFI44L* and *OAS2*, reflecting the known IFN-I signature in lupus. The MS subnetwork (Figure 6c) was dominated by *STAT1* and *CCR1*, consistent with their involvement in neuroinflammation. Comparative analysis confirmed that *STAT1, JAK2*, and *IRF8* were central across all subnetworks, underscoring their potential as pan-autoimmune therapeutic targets.

### Transcription factor and miRNA regulatory networks

**FIGURE 7:**
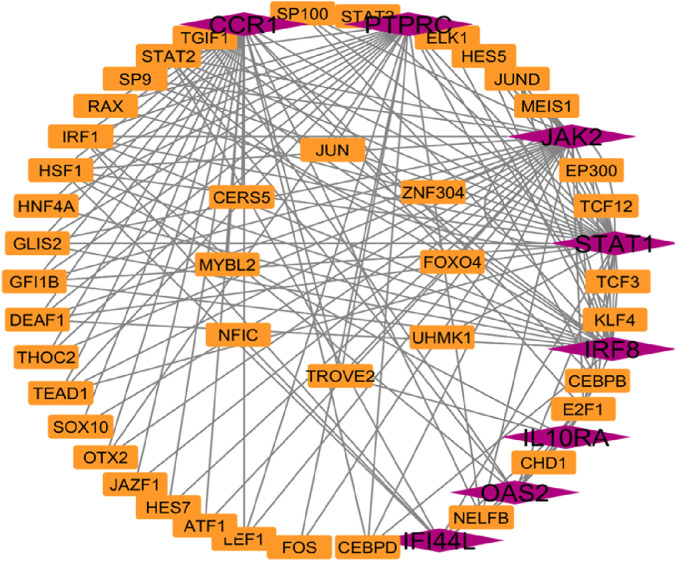
Transcription factors network analysis Hub genes are represent in pink colour and transcription factors are represented in orange colour.

**FIGURE 8:**
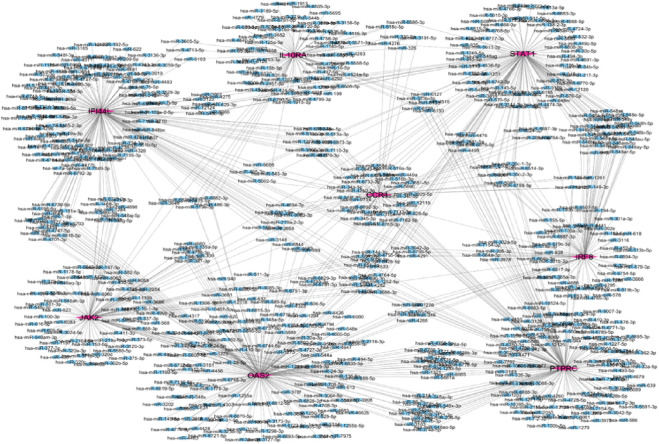
Hub gene and miRNA network the purple color represent hub genes and blue color represent in miRNA.

**FIGURE 9:**
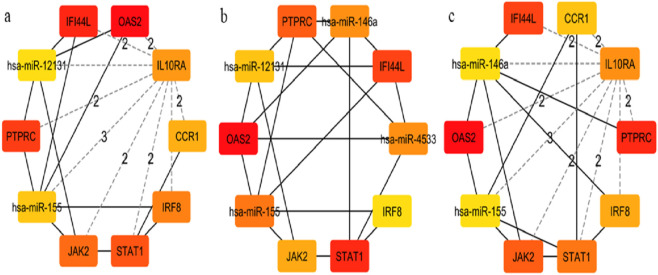
Hub-miRNA Network through topological analysis **(a)** Degree, **(b)** Closeness, **(c)** MCC used CytoHubba plugin to Cytoscape.

Network Analyst was used to construct TF and miRNA interaction maps for the hub genes. Transcriptional regulation by *IRF1, STAT2*, and NF-κB was prominent, as seen in Figure 7, Supplementary File. These TFs are known to regulate immune and interferon genes. Additionally, microRNAs such as miR-155 and miR-146a were identified as key post-transcriptional regulators (Figures 8, 9 Table 4 and Supplementary File). These miRNAs are highly conserved across species and frequently implicated in autoimmune regulation, offering additional therapeutic entry points.

**TABLE 4:** Top 10 miRNA topology analysis from three different ranking method methods.

S. No	Degree	Closeness	Betweenness
1	*OAS2*	*OAS2*	*OAS2*
2	*PTPRC*	*STAT1*	*PTPRC*
3	*STAT1*	*PTPRC*	*IFI44L*
4	*IFI44L*	*IFI44L*	*PTPRC*
5	*JAK2*	hsa-miR-155	*JAK2*
6	*IL10RA*	*PTPRC*	*STAT1*
7	*IRF8*	*JAK2*	*IL10RA*
8	*CCR1*	hsa-miR-12131	*IRF8*
9	hsa-miR-155	hsa-miR-4533	hsa-miR-155RF
10	hsa-miR-12131	*IRF8*	hsa-miR-146a

### Gene-disease and drug interaction network findings

**FIGURE 10:**
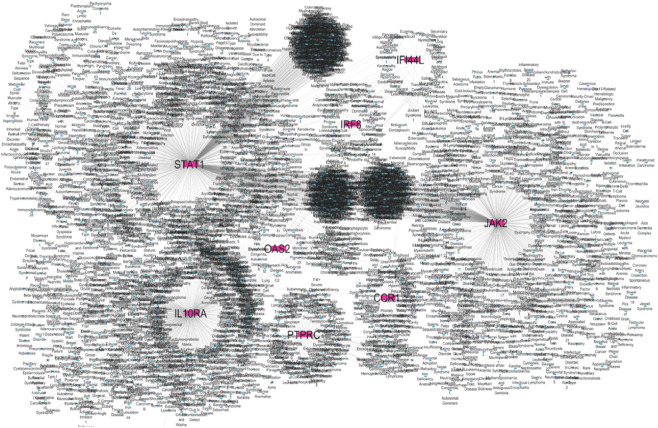
Gene–disease association hub genes are represented in pink colour and diseases are represented in blue colour.

**FIGURE 11:**
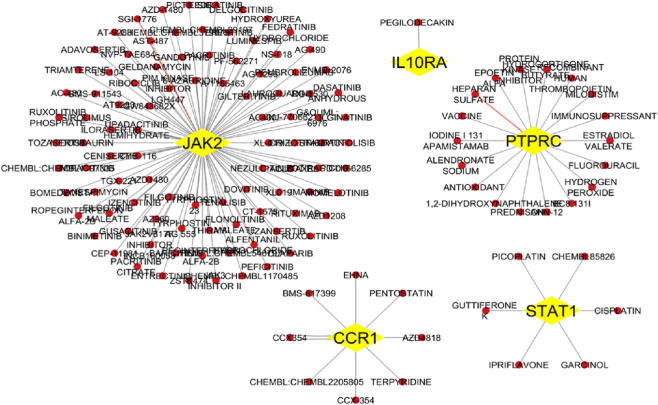
Drug-gene interaction network analysis hub gene are yellow colour and drugs are red colour.

DisGeNET confirmed the direct association of hub genes with RA, SLE, and MS, validating their disease relevance (Figure 10 and Supplementary File). DGIdb revealed that multiple hub genes were targeted by existing immunomodulatory drugs. For example, *JAK2* was associated with *JAK* inhibitors (baricitinib, tofacitinib), while natural compounds like luteolin and quercetin showed strong binding predictions for *OAS2* and *STAT1* (Figure 11 and Supplementary File). These interactions suggest potential for repurposing approved drugs or combining them with nutraceuticals for improved autoimmune therapy.

### Molecular docking indicates high affinity interactions

**FIGURE 12:**
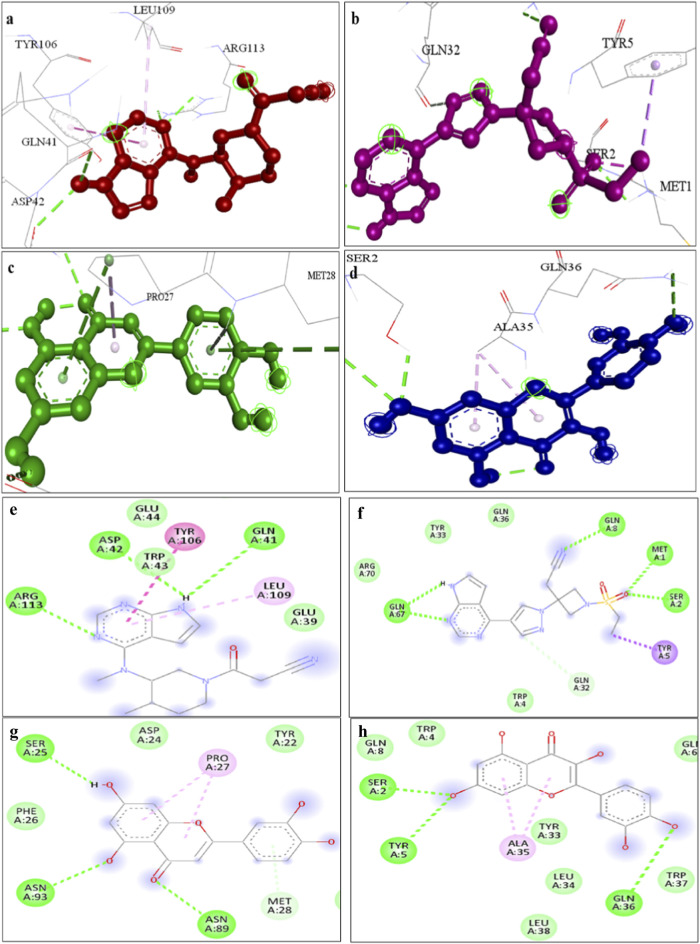
A representation of 3D interaction analysis of quercetin the top first docked complexes. Interaction of **(a)** Tofacitinib **(b)** Baricitinib, **(c)** Luteolin, **(d)** Quercetin with STAT1 protein and 2D interaction of **(e)** Tofacitinib **(f)** Baricitinib, **(g)** Luteolin, **(h)** Quercetin with *STAT1* protein.

Molecular docking simulations were conducted using Auto Dock Vina. Among the compounds tested, luteolin exhibited the highest binding affinity to *STAT1* (−9.1 kcal/mol), while quercetin bound strongly to OAS2 (−8.7 kcal/mol) (Table 5). Drug likeness properties of the ligands are presented in Table 6. These interactions involved key residues in the DNA-binding and SH2 domains, critical for protein activation. Figure 12 illustrates both the 3D and 2D interaction maps, highlighting multiple hydrogen bonds and hydrophobic interactions that contribute to stability. These findings suggest luteolin and quercetin as promising lead compounds.

**TABLE 5:** Protein *STAT1* and ligand four compounds with interaction residues.

S. No	*STAT1* and ligand	Binding affinity (kcal/mol)	H-bond interaction	Other interactions
1.	Baricitinib	−7.2	Asp 42, Gln 41, Arg 113	Tyr 106, Leu 109
2.	Tofacitinib	−5.6	Met 1, Ser 2, Gln 8, Gln 67	Tyr 5
3.	Luteolin	−9.1	Ser25, Asn 93, Asn89	Pro 27
4.	Quercetin	−7.8	Ser 2, Tyr 5, Gln 36	Ala 35

**TABLE 6:** Drug-likeness properties of the selected ligands.

S. No	Ligand	MW g/mol	HBD	HBA	Log p (<5)	TPSA Å2	nrotb	nViol
1.	Baricitinib	286.24	4	6	1.86	111.13	1	0
2.	Tofacitinib	371.4	1	7	1.38	128.94	5	0
3.	Luteolin	312.37	1	4	1.70	88.91	4	0
4.	Quercetin	302.24	5	7	1.63	131.36	1	0

### Molecular dynamics simulation validates complex stability

**FIGURE 13:**
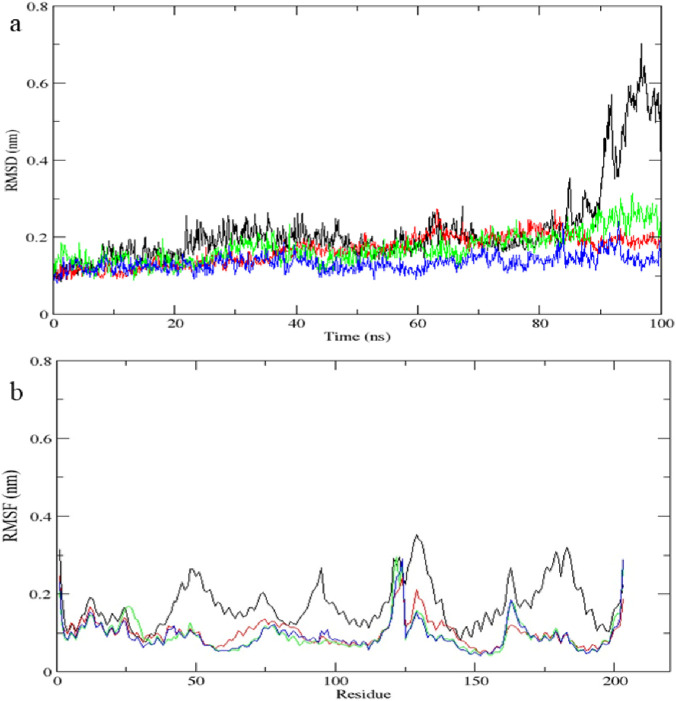
Molecular dynamics simulation results of complex native protein *STAT1* with ligand complex with Tofacitinib (black), complex baricitinib (red), complex luteolin (green), and complex Quercetin (blue). **(a)** Time-dependent RMSD of c-a backbone **(b)** The RMSF of c-a atoms.

**FIGURE 14:**
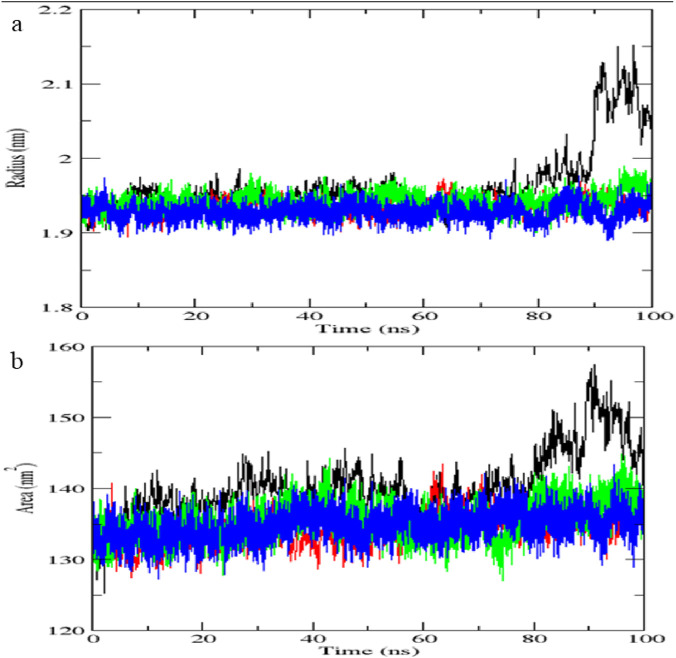
**(a)** Radius of gyration vs. time. **(b)** SASA vs. time.

**FIGURE 15:**
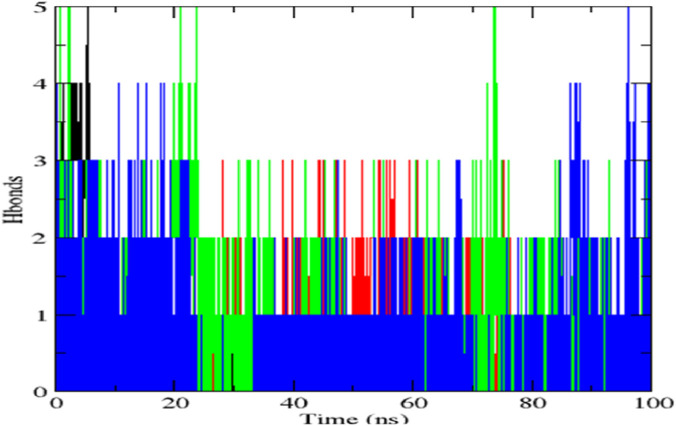
Hydrogen bonds formation vs. time.

**FIGURE 16:**
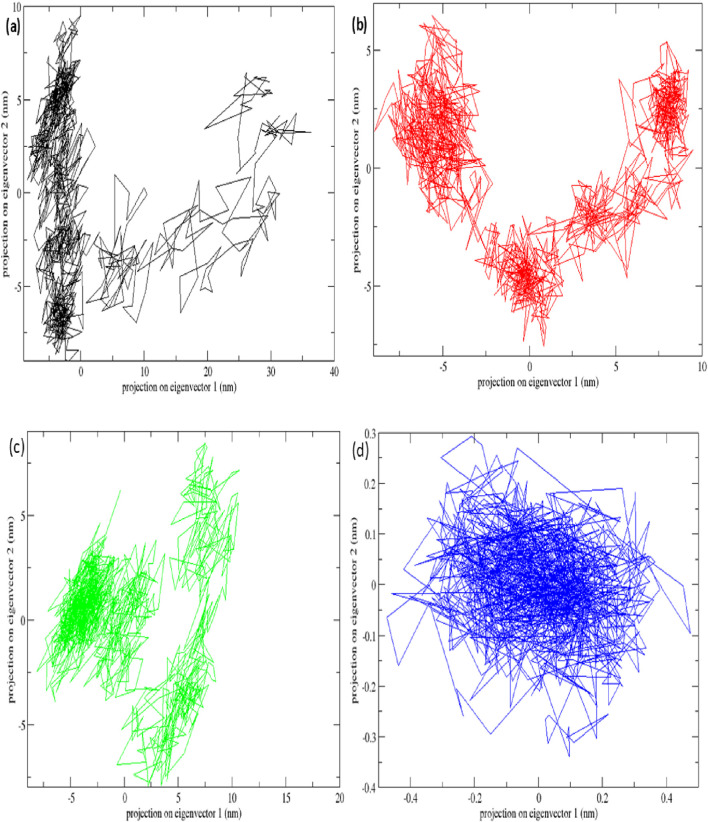
Principal component analysis plots of all four complexes and *STAT1* protein with **(a)** complex Baricitinib (black), **(b)** complex Tofacitinib (red), **(c)** complex luteolin (green) and **(d)** complex quercetin (blue).

The *STAT1*-luteolin complex was subjected to a 100 ns molecular dynamics simulation in GROMACS. RMSD and RMSF analysis (Figure 13) confirmed structural stability of the complex, with minimal fluctuations. The radius of gyration (Figure 14) and solvent-accessible surface area profiles remained consistent throughout the simulation. Hydrogen bond analysis (Figure 15) showed sustained interactions. Principal component analysis (Figure 16) baricitinib, tofacitinib complex occupies a large space and luteolin, quercetin complex occupies lesser space and MM-PBSA calculations (Table 7) yielded a total binding energy of −45.4 kcal/mol. Together, these metrics validate the stability and potential efficacy of luteolin as an inhibitor of *STAT1*.

**TABLE 7:** Molecular mechanics poisson–boltzmann surface area binding energies of (kJ/mol) of complexes.

Protein-ligand complex	ΔVDWaals (kJ/mol)	ΔEEL (kJ/mol)	ΔGGAS (kJ/mol)	ΔGSOLV (kJ/mol)	ΔG Total (kJ/mol)
*STAT1*-baricitinib	−6.16 ± 0.22	−6.07 ± 1.50	−12.23 ± 1.52	8.43 ± 0.94	−3.8 ± 1.79
*STAT1*-tofacitinib	−16.32 ± 0.36	−11.55 ± 1.73	−27.87 ± 1.77	20.49 ± 0.57	−7.38 ± 1.86
*STAT1*-luteolin	−26.18 ± 0.80	−18.54 ± 0.40	−45.43 ± 0.90	27.43 ± 1.50	−18 ± 1.75
*STAT1*-quercetin	−15.4 ± 0.80	−6.17 ± 0.40	−21.57 ± 0.90	12.82 ± 1.50	−8.72 ± 1.75

### Visualization of multi-layered network results

**FIGURE 17:**
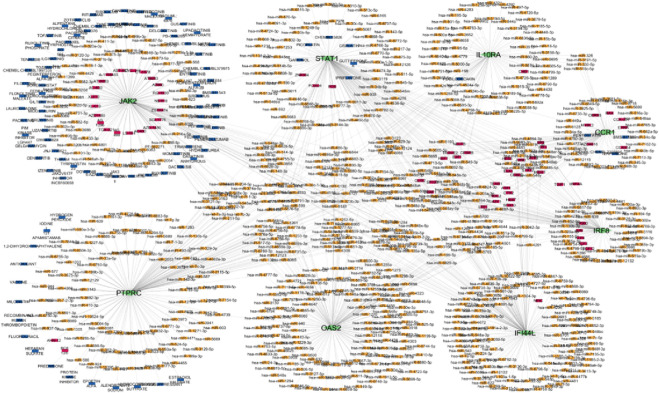
Multi layered network hub genes green colour, miRNA orange colour, TF pink colour, drugs blue colour.

Integrated visualization was performed to synthesize results from differential expression, PPI, regulatory, and drug interaction analyses, displayed consistent expression patterns of hub genes across diseases. A unified network (Figure 17) was constructed to show interactions among TFs, miRNAs, hub genes, and drugs. This systems-level perspective highlights convergence on a few central regulators, supporting their prioritization as universal autoimmune targets.

## Discussion

In the present investigation, we undertook a combined systems biology approach to decipher the common molecular characteristics of Rheumatoid Arthritis (RA), Systemic Lupus Erythematosus (SLE), and Multiple Sclerosis (MS). Although these diseases manifest in distinct physiological systems joints, systemic organs, and the central nervous system we hypothesized that they share a fundamental biological origin. By integrating transcriptomic data across these conditions, we successfully identified shared pathogenic drivers and prioritized novel therapeutic targets.

We observed that despite the heterogeneity in clinical presentation, these diseases converge on a highly conserved immune-inflammatory signature. Our pathway analysis clearly indicates that the Type I Interferon (IFN-I) and *JAK-STAT* signalling pathways are the primary engines driving this shared pathogenesis. This observation is in strong agreement with recent studies; for instance, Shen and You (2025) recently demonstrated that RA and SLE share significant immune regulatory genes like *IFIT3* and *TNFSF13B*, which are directly linked to Type I interferon signalling. Furthermore, Naveed et al. (2025) reported that shared genetic linkages in autoimmune diseases frequently cluster around these specific inflammatory cascades, validating our findings.

An interesting observation in our study concerns Siglec-1. While we initially noted Siglec-1 as a biomarker for interferon activity (York et al., 2007), it did not appear as a top hub gene in our network. This does not imply that Siglec-1 is insignificant; rather, it suggests that our computational method successfully prioritized the “master regulators” upstream drivers like *STAT1* over the “downstream products” biomarkers. Consequently, we identified eight key hub genes: *STAT1, PTPRC, IRF8, JAK2, IL10RA, OAS2, CCR1,* and *IFI44L*. Among these, we selected *STAT1* as the most critical drug target due to its centrality in the protein-protein interaction network.

A major finding of our study is the identification of Luteolin, a natural phytocompound, as a potent inhibitor of *STAT1*. We performed molecular docking studies to compare Luteolin with standard FDA-approved drugs. The results were highly encouraging. We found that Luteolin showed a binding affinity of −9.1 kcal/mol, which is significantly superior to the commercially available drugs baricitinib (−7.2 kcal/mol) and tofacitinib (−5.6 kcal/mol). This finding is supported by recent literature; Nadalin et al. (2024), Xia et al. (2016) experimentally proved that Luteolin alleviates apoptosis and inflammation by directly inhibiting the *JAK/STAT* signalling pathway (Ren et al., 2024; Guo F. et al., 2024). Additionally, Frontiers in Immunology (2025) published a review highlighting that flavonoids like Luteolin effectively modulate macrophage polarization and block NF-κB and *JAK-STAT* signals in metabolic and autoimmune disorders (Wang et al., 2025).

To further validate this, we ran a 100 ns molecular dynamics simulation. The complex remained stable throughout the simulation period, confirming that Luteolin can effectively bind and block the *STAT1* pathway. This aligns with the work of Peng et al. (2024), who reported that Luteolin significantly reduces the secretion of pro-inflammatory factors such as TNF-α and IL-6, further proving its efficacy as an immunomodulator.

It is also noteworthy that while the diseases share a common core, they retain unique characteristics. We observed that the RA network was enriched with genes for joint inflammation, SLE showed a strong interferon signature, and MS emphasized neuroinflammation. This supports the common core, unique periphery model, suggesting that while broad-spectrum agents (like Luteolin) can target the shared *STAT1* core, disease-specific therapies are still necessary for unique symptoms.

We acknowledge that the present study has certain limitations. Since this is an *in silico* work, the findings need to be validated through *in vitro* and *in vivo* experiments. However, the alignment of our results with the recent wet-lab findings of Ren et al. (2024) and Shen and You (2025) gives us high confidence in our predictions.

In conclusion, our study provides strong evidence that RA, SLE, and MS share a common molecular mechanism driven by *STAT1* and Interferon signalling. We have demonstrated that Luteolin has excellent potential as a lead molecule to target *STAT1*, showing better theoretical efficacy than some existing synthetic drugs. These findings pave the way for developing cost-effective, broad-spectrum therapeutics for autoimmune diseases.

## Conclusion

Our meta-analysis of transcriptome data from patients with RA, MS, and SLE identified a core set of common differentially expressed genes. Through functional enrichment analysis, we narrowed this list to eight key hub genes: *STAT1, CCR1, JAK2, IRF8, OAS2, IFI44L, IL10RA*, and *PTPRC*. These genes are central to the shared pathology of these diseases, and our study also explored their complex regulatory networks. To investigate their therapeutic potential, we performed molecular docking and dynamics simulations. Interestingly, this analysis showed that the natural compounds Luteolin and Quercetin are strong candidates for new treatments, performing comparably to established drugs such as baricitinib and tofacitinib. The clear takeaway is that these eight genes are valuable clinical targets, offering a new direction for developing targeted therapies for these autoimmune conditions.

## Data Availability

The original contributions presented in the study are included in the article/Supplementary Material, further inquiries can be directed to the corresponding author.
